# Effects of apple cider vinegar on glycemic control and insulin sensitivity in patients with type 2 diabetes: A GRADE-assessed systematic review and dose–response meta-analysis of controlled clinical trials

**DOI:** 10.3389/fnut.2025.1528383

**Published:** 2025-01-30

**Authors:** Donya Arjmandfard, Mehrdad Behzadi, Zahra Sohrabi, Mohsen Mohammadi Sartang

**Affiliations:** ^1^Student Research Committee, School of Nutrition and Food Sciences, Shiraz University of Medical Sciences, Shiraz, Iran; ^2^Nutrition Research Center, School of Nutrition and Food Sciences, Shiraz University of Medical Sciences, Shiraz, Iran

**Keywords:** Apple cider vinegar, FBS, HbA1c, T2DM, meta-analysis

## Abstract

**Background and aims:**

Diabetes mellitus (DM) is a multifactorial metabolic disorder that affects the body’s ability to regulate blood sugar levels. Apple cider vinegar (ACV) could possibly improve diabetes; nevertheless, evidences provide conflicting results. This study aimed to evaluate the effects of ACV on glycemic profile in type 2 diabetes patients (T2DM) in controlled trials (CTs) by systematically reviewing and dose–response meta-analysis.

**Methods:**

The Scopus, PubMed, and Web of Science databases were searched until November 2024 according to a systematic approach. All CTs investigating ACV’s effects on glycemic factors were included. We used a random-effects model to calculate WMDs and 95% confidence intervals (CIs). The present study assessed publication bias, sensitivity analysis, meta-regression, and heterogeneity based on standard methods. We assessed the bias risk of the included studies using Cochrane quality assessments and used GRADE (Grading of Recommendations Assessment, Development, and Evaluation) to calculate evidence certainty. We registered the study protocol at Prospero (no. CRD42023457493).

**Results:**

Overall, we included seven studies in this meta-analysis. ACV significantly reduced fasting blood sugar (FBS) (WMD: −21.929 mg/dL, 95% CI: −29.19, −14.67, *p* < 0.001) and HbA1c (WMD: −1.53, 95% CI: −2.65, −0.41, *p* = 0.008) and increased insulin (WMD: 2.059 μu/ml, 95% CI: 0.26, 3.86, *p* = 0.025), while it did not affect hemostatic model assessment for insulin resistance (HOMA-IR). We observed linear and non-linear associations between ACV consumption and FBS levels (*p* < 0.001). Each 1 mL/day increase in ACV consumption was associated with a-1.255 mg/dL reduction in FBS. Moreover, greater effects on FBS were in dosages >10.

**Conclusion:**

ACV had positive effects on FBS and HbA1c in T2DM patients.

**Systematic Review Registration:**

The study protocol was registered at Prospero (no. CRD42023457493).

## 1 Introduction

Diabetes mellitus (DM) is a multifactorial metabolic disorder that affects the body’s ability to regulate blood sugar levels (1). Type 2 diabetes mellitus (T2DM) is a condition characterized by hyperglycemia due to inadequate insulin secretion and insulin resistance (2). More than 500 million people worldwide suffer from diabetes, and by the year 2045, this number is expected to reach 783 million (3). About 90% of all diabetes patients have T2DM. There are several secondary complications associated with it, including cardiovascular disease, strokes, and diabetic retinopathy (4–6). A growing concern has been raised because an increase in T2DM prevalence will result in an increase in chronic and acute diseases in general. This will have profound effects on the quality of life, economic expenses and demand for health care services (7).

The treatment of T2DM relies on the long-term use of anti-diabetic drugs (8, 9), as there is no final cure for the disease (10, 11). It has been demonstrated that dietary modifications are crucial to successfully achieving and maintaining glycemic targets for people with type 2 diabetes mellitus (T2DM) and optimizing their health outcomes (12, 13). Therefore, it is imperative that new methods be explored that may delay or even reverse the progression of T2DM.

The use of plants and their derivatives in contemporary research and practice has gained much attention due to their beneficial effects on controlling glycemic control (14, 15). In this regard, vinegar is among the most commonly used plant derivatives. One of the most common types of vinegar is apple cider vinegar (ACV), which is made by fermenting apples (16). As a preservative agent and flavoring in foods, this acidic solution is used worldwide (17). There are several flavonoids in ACV, such as catechin, ferulic acid, caffeic acid and gallic acid which can improve glucose metabolism (18, 19). These components have been shown to play roles in glucose metabolism and possess anti-inflammatory and antioxidant properties. While acetic acid is indeed the primary active ingredient in all kinds of vinegar, the synergistic effects of these additional compounds present in ACV make it distinct and particularly relevant for investigating glycemic control in T2DM.

Animal studies have revealed that ACV has a number of pharmacological functions, including anti-inflammatory, anti-oxidant, anti-diabetic, anti-hyperlipidemic, and anti-hypertensive effects (20–23). There have been several randomized controlled trials (RCTs) conducted in this field. A contradictory effect was observed on glycemic indexes as a result of these interventions (24–30). In a meta-analysis conducted by Hadi et al., in 2021, on 9 RCTs, they almost reached positive conclusions about the effect of ACV on lipid and glycemic profiles in adults with various health conditions including diabetes, obesity, overweight, and the like (31).

Therefore, this systematic review and dose–response meta-analysis aimed to pool the results of various related controlled trials (CTs) assessing the effects of ACV on glycemic indices and insulin sensitivity in patients with T2DM.

## 2 Methods

### 2.1 Search strategy

The present study was conducted according to PRISMA guidelines (Preferred Reporting Items for Systematic Reviews and Meta-Analyses) (32). In addition, we used Prospero to register the study protocol (no. CRD42023457493). To identify relevant CTs that investigated whether ACV influenced the glycemic profile of T2DM patients, the current study conducted a comprehensive systematic search in the online databases Scopus, PubMed, and Web of Science until November 2024. No limitations were applied to the date or language of the studies. A detailed description of the database search strategy can be found in Table S1. Furthermore, we also reviewed relevant meta-analyses and reviews in addition to hand-searching reference lists. For notification of new publications, email alerts were also set up using PubMed’s “My NCBI” (National Center for Biotechnology Information), Scopus, and Web of Science.

### 2.2 Study selection

Two independent investigators (DA and MB) reviewed the articles. To resolve discrepancies, discussions were held and conflicting opinions were resolved by consulting a third author (ZS) if the authors could not reach a consensus. We selected the studies for analysis according to the following criteria (Table 1): (1) controlled clinical trials featuring either a crossover or parallel design; (2) ACV’s effect on glycemic profile could be extracted from the article (glycemic indices at baseline and follow-up were available with standard deviations (SD), standard errors (SEs), and 95% confidence intervals (CIs) for both control and intervention groups); (3) we distinguished the Control and intervention groups only by the ACV in a controlled study; (4) the intervention should last at least 2 weeks; (5) adults (18 years of age or older) with type 2 diabetes participated in the study.

**Table 1:** PICOS criteria for inclusion and exclusion of studies.

Parameter	Criteria
Participant	Type 2 diabetes mellitus patients
Intervention	Apple cider vinegar
Comparator	Placebo
Outcomes	FBS, HbA1c, HOMA-IR, Insulin
Study design	Controlled clinical trial

FBS, fasting blood sugar; HbA1c, glycated hemoglobin; HOMA-IR, homeostasis model assessment for insulin resistance.

Following is the list of exclusion criteria for studies: (i) the net effects of ACV could not be determined; (ii) the duration of intervention was <2 weeks; (iii) studies that were semi-experimental, cohort, case–control, and cross-sectional designs, review articles, and ecological studies; (iv) data on baseline and follow-up glycemic parameters were insufficient.

### 2.3 Data extraction

Two authors (DA and MB) selected the eligible articles independently by based on screening forms for inclusions and exclusions. We extracted the data using an Excel form for each article. This document has been revised to reflect the current title as well as the following abstracted information: It includes the first author’s name, the location of the study, the publication year, the design of the study as parallel or crossover, the number of participants in each group, dosages and types of intervention and control, durations of the interventions, the health status of the participants, and demographic information such as age and gender. Moreover, we extracted the mean values and standard deviations of glycemic parameters at baseline as well as at the end of the study. For trials that included multiple measurements, only the final values were considered for analysis.

### 2.4 Quality assessment

Two authors (MS and ZS) independently assessed the bias risk of the included studies using the last version of Cochrane quality assessment tools by Higgins that contain seven domains (33). We assigned a “high risk” score to every domain if there were methodological deficiencies that could have affected the results. In the case of no defects being found in those domains, the domain received a “low risk” score, and in the event of insufficient information available, it received an “unclear risk” score. Those studies that scored “low risk” in any of the domains were considered high-quality and had a completely low bias risk.

We used Grading of Recommendations Assessment, Development, and Evaluation (GRADE) to assess and summarize the total quality of the evidence of all studies. GRADE is a methodologically strong and clear approach for judging the strength of recommendations and the certainty of evidence. This method includes four key components: assessing the quality of evidence, evaluating the balance between benefits and harms, considering values and preferences, and making explicit judgments about the strength of recommendations (34).

### 2.5 Statistical analysis

This meta-analysis was conducted using Comprehensive Meta-Analysis (CMA) V3 software (35). When the probability value (*p*-value) was <0.05, it was considered statistically significant. For all parameters, we used a random effects model and assessed the effects of ACV on the following outcomes: (i) fasting blood sugar (FBS), (ii) parameter of insulin resistance including homeostasis model assessment for insulin resistance, (HOMA-IR), (iii) and quantitative insulin sensitivity checks index (QUICKI), (iv) serum insulin levels and (v) glycated hemoglobin (HbA1c). We expressed the effect sizes by weighted mean differences (WMD) and 95% CI. The mean and SD of glycemic values were calculated in both the ACV and control groups before and after the intervention to calculate net changes: Trial end value - trial baseline value. We also calculated the mean difference as follows: (final value in the ACV group – baseline value in the ACV group) - (final value in the control group - baseline value in the control group). If no SD was reported, it was calculated as follows: SD = square root [(SD pre-intervention)^2^ + (SD post-intervention)^2^ - (2 R × SD pre-intervention × SD post-intervention)] (36). We used the following formula for calculating SD from standard error of the mean (SEM) in some studies: SEM to SD. SDs = SEs × square root (n), where n refers to the number of individuals in each group. In order to estimate medians, ranges, and 95% confidence intervals, the authors of the current study used Hozo et al.’s method (37). We used the Get Data Graph Digitizer software to extract the data (38) form the results in the form of graphs. Statistical evaluation of heterogeneity was performed using Cochran’s *Q*-test with significance set at 0.1 and the I^2^ test to calculate the percentage of heterogeneity (I^2^ value ≥50% indicates significant heterogeneity). In order to assess how each trial impacted the overall effect size, we conducted a sensitivity analysis using the leave-one-out method (39). In order to determine the influence of factors including dose, duration, and design of CTs, sub-group analysis was performed. In the current study, the authors also used meta-regressions to assess the association between moderating variables, including dose and duration of the intervention, and effect sizes. Crippa et al. (40) suggestion was used to analyze the dose–response effect of ACV intakes on FBS among people with T2DM. We performed a dose–response analysis using the command “drmeta” in Stata, version 17 (StataCorp, Texas, USA).

To assess the publication bias, we used the funnel plot, in addition to Begg’s rank correlation and Egger’s weighted regression analysis. To adjust for publication bias, “trim and fill” and “fail-safe N” methods of Duval and Tweedie (41) were applied.

## 3 Results

### 3.1 Findings from the systematic search

**Figure 1:**
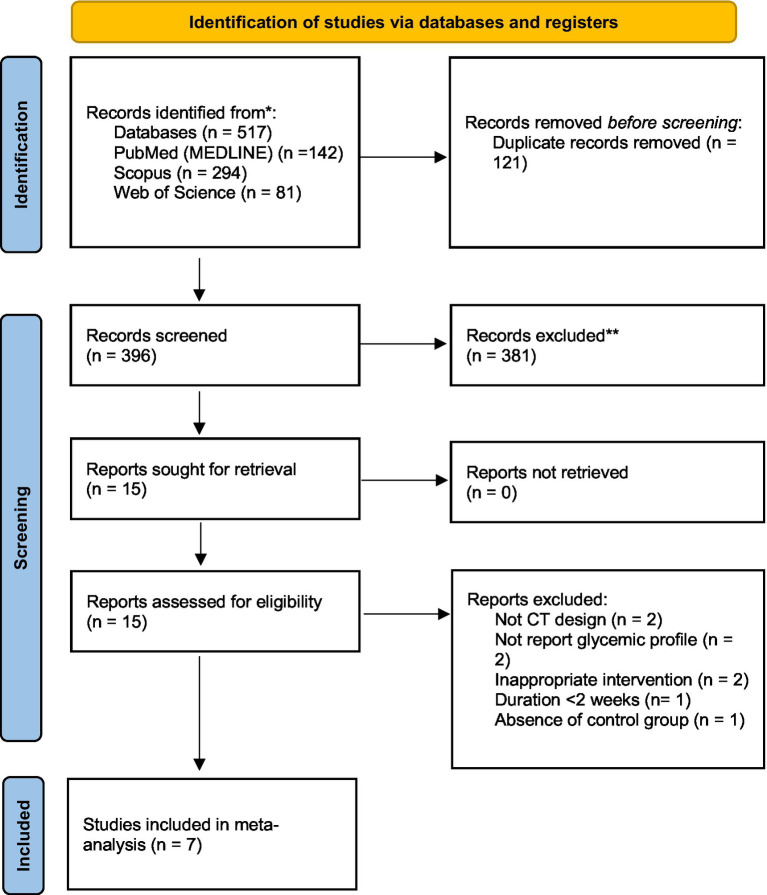
PRISMA 2020 flow diagram of databases searches, registers and other sources.

According to the results of the initial search in the online databases, we found 517 articles. Among them, 121 papers were duplicates; therefore, they were excluded. Based on the titles and abstracts of the remaining articles, 381 were determined to be irrelevant. Thus, the full-text assessment consisted of 15 papers. Of the 15 articles, two were excluded for being non-CTs (42, 43). Additionally, two CTs did not measure the desired outcomes (18, 44). As an intervention, two CTs used a type of apple not considered in the analysis (45, 46). The study of Mousavi et al. (47) was excluded because of the short duration (<2 weeks intervention duration). The study of Heljić et al. (48) was also excluded due to the absence of a control group. Finally, seven studies were included in the current systematic review and meta-analysis with complete findings (24–30) (Figure 1).

### 3.2 Characteristics of included studies

In total, we randomly assigned 463 participants to 7 qualified studies (235 to the ACV group, and 228 to the control group) (Table 2). These trials had participants ranging from 38 (24) to 110 (28). The included studies were published between the years 2009 and 2023. The studies were done in Iran (five studies) (24, 25, 27, 29, 30), Tunisia (26) and Pakistan (28). The mean age of the participants ranged from 49.2 (30) to 54.6 (24) years old. All studies were conducted on both men and women except Halima et al. (26) study which did not report. Intervention duration ranged from 4 (26, 29) to 12 (28) weeks. All studies had parallel designs. Of the seven studies, one was single-blinded (28), two were double-blinded (26, 29), and four did not do blinding (24, 25, 27, 30). In all included studies, the ACV used was produced with the same procedure containing almost 5% acetic acid.

**Table 2:** Demographic characteristics of the included studies.

Author (Year)	Design	Country	Patient status	Sample size (intervention/control)	Age (years) (intervention/control)	Gender	Duration (weeks)	Intervention/control (type and dosage)	Registered	Funding
Ebrahimi-Mamaghani et al. (24)	Rn/Pa	Iran	Type 2 diabetes	19/19	54.6/53.8	Both	8	13.75 mL/d ACV/−	Yes	Investigator-initiated
Mahmoodi et al. (29)	Db/Pa	Iran	Type 2 diabetes	30/30	NR	Both	4	15 mL/d ACV/water	Yes	NR
Halima et al. (26)	Db/Rn/Pa	Tunisia	Type 2 diabetes	24/20	NR	NR	4	15 mL/d ACV/water	Yes	NR
Mohammadpourhodki et al. (30)	Rn/Pa	Iran	Type 2 diabetes	38/38	49.2/49.2	Both	8	20 mL/d ACV/−	Yes	NR
Gheflati et al. (25)	Rn/Pa	Iran	Type 2 diabetes	32/30	49.47/52.1	Both	8	20 mL/d ACV/P	Yes	Investigator-initiated
Kausar et al. (28)	Sb/Rn/Pa	Pakistan	Type 2 diabetes	55/55	51.1/50.4	Both	12	15 mL/d ACV/P	Yes	Investigator-initiated
Jafarirad et al. (27)	Rn/Pa	Iran	Type 2 diabetes	37/36	53.11/52.94	Both	8	30 mL/d ACV + dietary recommendation/ dietary recommendation	Yes	Investigator-initiated

Apple Cider Vinegar; NR, Not Reported; Db, Double-blinded; Sb, Single-blinded; Rn, Randomized; Pa, Parallel; P, Placebo.

### 3.3 Data quality

Table 3 summarizes the results of Cochrane’s risk of bias tool for the quality assessment of studies. Five trials were classified as low quality (high bias risk in >2 domains) (24, 25, 27, 28, 30), one trial was classified as moderate quality (high bias risk in 2 domains) (29) and one was classified as high quality (high bias risk in <2 domains) (26). Evidences for FBS and insulin were moderate GRADE while for HbA1c and HOMA-IR were low GRADE (Table 4).

**Table 3:** Results of risk of bias assessment for CTs included in the current meta-analysis on the effect of apple vinegar supplementation on glycemic control in patients with type 2.

Study	Sequence generation	Allocation concealment	Selective outcome reporting	Other sources of bias	Blinding of participants and personnel	Blinding of outcome assessment	Incomplete outcome data	Overall risk of bias
Ebrahimi-Mamaghani et al. (24)	U	U	H	L	H	H	L	H
Mahmoodi et al. (29)	H	U	H	L	L	U	L	M
Halima et al. (26)	U	U	H	L	L	U	L	L
Mohammadpourhodki et al. (30)	U	U	H	L	H	H	L	H
Gheflati et al. (25)	L	U	H	L	H	H	L	H
Kausar et al. (28)	L	U	H	L	H	H	L	H
Jafarirad et al. (27)	L	H	L	L	H	H	L	H

U, unclear risk of bias; L, low risk of bias; H, high risk of bias; M, moderate risk of bias.

**Table 4:** GRADE.

Certainty assessment							No. of patients		Effect	Certainty	Importance
No. of studies	Study design	Risk of bias	Inconsistency	Indirectness	Imprecision	Other considerations	(ACV)	(placebo)	Absolute (95% CI)		
FBS											
7	Randomized trials	Serious^a^	Not serious	Not serious	Not serious	None	235	228	MD 21.929 mg/dL lower (29.19 lower to 14.67 lower)	⨁⨁⨁◯ Moderate	IMPORTANT
HbA1c											
4	Randomized trials	Serious^b^	Serious^c^	Not serious	Not serious	None	160	159	MD 1.53 mg/dL lower (2.65 lower to 0.41 lower)	⨁⨁◯◯ Low	IMPORTANT
HOMA-IR											
3	Randomized trials	Serious^d^	Not serious	Not serious	Serious^e^	None	88	85	MD 0.631 mg/dL higher (0.99 lower to 2.25 higher)	⨁⨁◯◯ Low	IMPORTANT
Insulin											
3	Randomized trials	Serious^f^	Not serious	Not serious	Not serious	None	88	85	MD 2.059 μu/ml higher (0.26 higher–3.86 higher)	⨁⨁⨁◯ Moderate	IMPORTANT

FBS, fasting blood sugar, HbA1c, glycated hemoglobin; HOMA-IR, homeostasis model assessment for insulin resistance; CI, confidence interval; MD, mean difference. ^a^Serious risk of bias since 4 trials were at high risk of bias. Downgraded. ^b^Serious risk of bias since 2 trials were at high risk of bias. Downgraded. ^c^Serious inconsistency since I^2^ = 88.61%. Downgraded. ^d^Serious risk of bias since all trials were at high risk of bias. Downgraded. ^e^Serious imprecision since the result of the analysis is not meaningful. Downgraded. ^f^Serious risk of bias since all trials were at high risk of bias. Downgraded.

### 3.4 Meta-analysis

**Figure 2:**
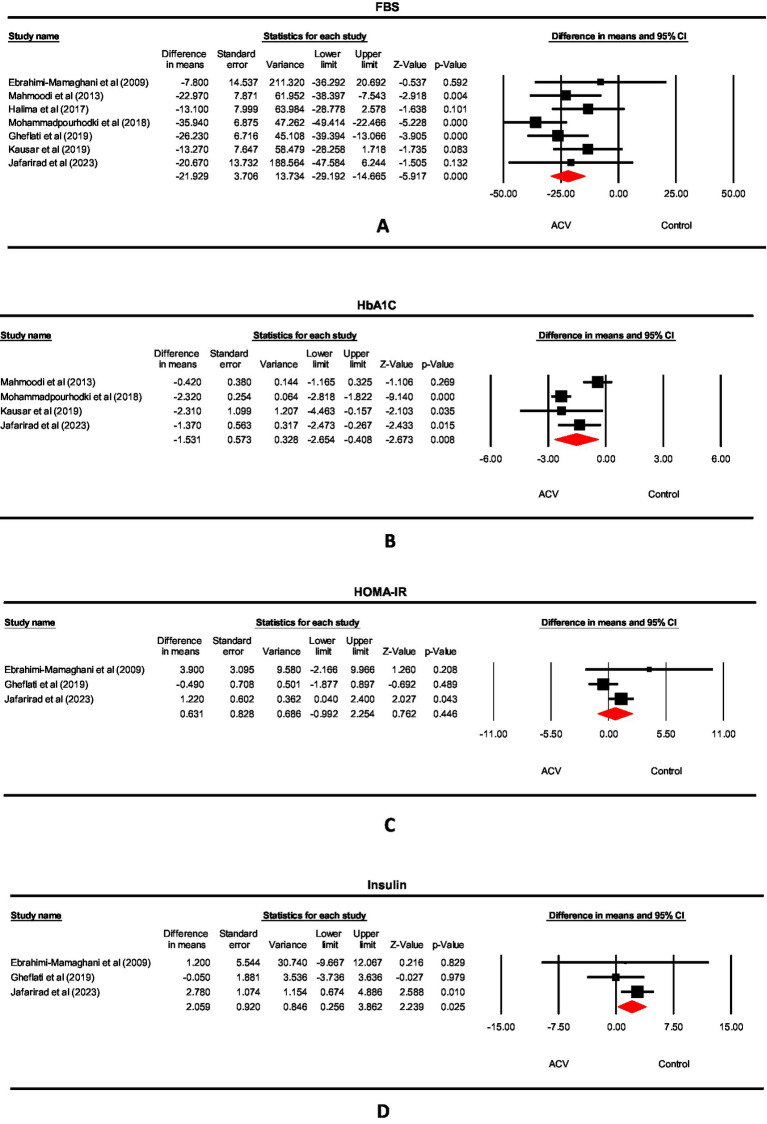
Forest plot for the effect of ACV on **(A)** FBS, **(B)** HbA1c, **(C)** HOMA-IR and **(D)** Insulin in T2DM patients, expressed as mean differences between intervention and control groups.

The forest plots of FBS, HbA1c, HOMA-IR, and insulin levels from the meta-analysis are shown in Figures 2A–D. Just one study measured QUICKI; we could not perform a meta-analysis on this parameter. Because of the low number of studies, we have done subgroup analysis, meta-regression, and dose–response only for FBS.

#### 3.4.1 Meta-analysis on ACV and FBS

**Figure 3:**
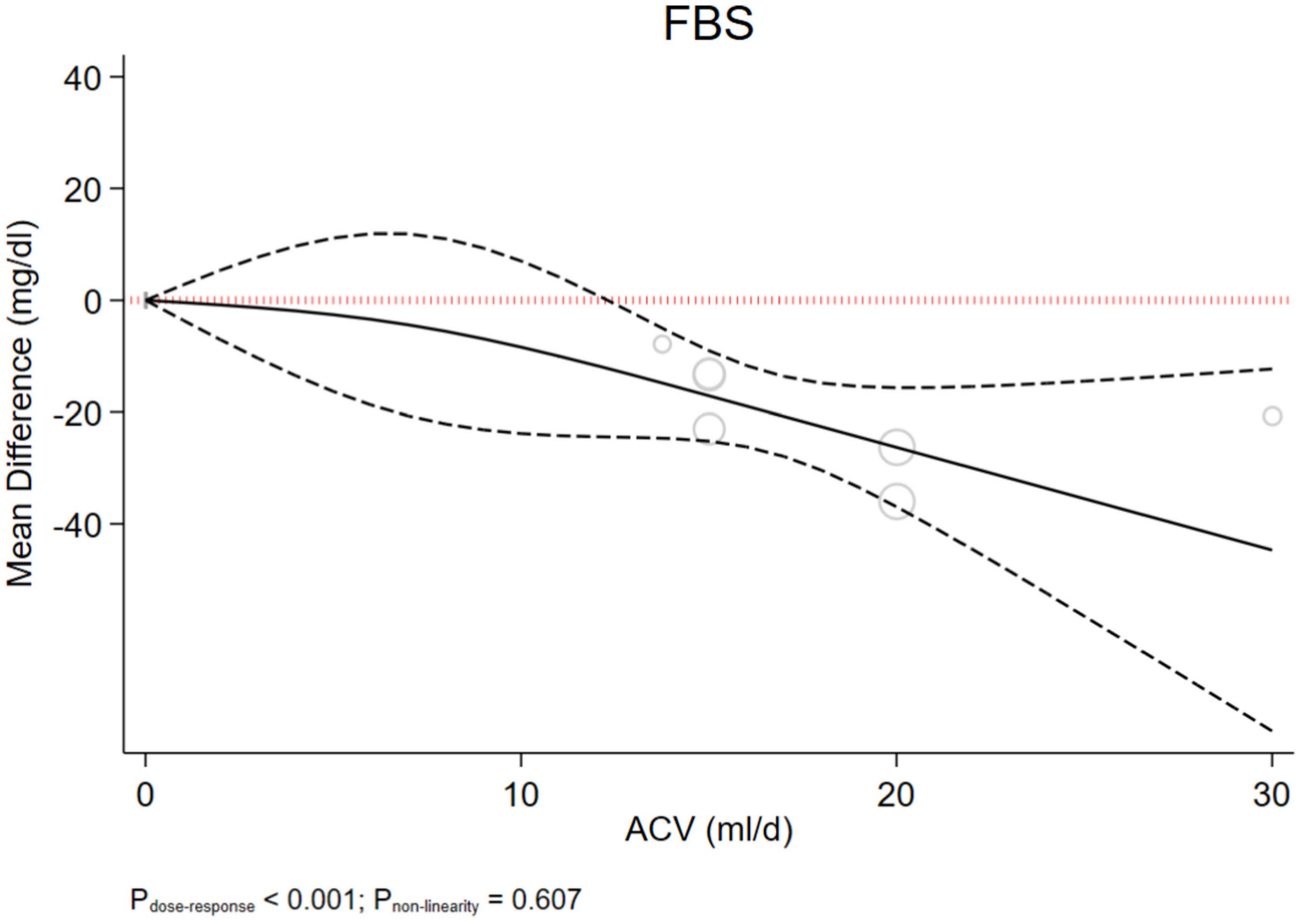
Non-linear dose–response effects of ACV dosages (ml/d) on FBS, in T2DM patients. The 95% CI is demonstrated in the dashed line.

We included seven studies involving 463 participants. ACV significantly reduced FBS based on the results of a random-effect model (WMD: −21.929 mg/dL, 95% CI: −29.19, −14.67, *p* < 0.001), with non-significant heterogeneity (I^2^ = 20.11%, *p* = 0.237) (Figure 2A). We summarized the results of the subgroup analysis in Table 5. Considering the sub-group analysis based on the dose and duration, significant effects were observed in dosages>15 g/d, and durations ≥8 weeks. However, according to meta-regression, we found no significant association between changes in FBS values with ACV dose (*p* = 0.184) and duration (*p* = 0.928) (Figures S2A,B). On the other hand, the dose–response meta-analysis of ACV intake and changes in FBS included 7 studies. The present study found a significant linear association between ACV consumption and changes in FBS so that each 1 mL/day increase in ACV consumption was associated with a-1.255 mg/dL reduction in FBS (*p* < 0.001). Moreover, the results showed a non-linear association, in which, a significant reduction in FBS was seen in dose >10 mL/d (P_dose–response_ < 0.001, P_non-linear_ = 0.607) (Figure 3).

**Table 5:** Results of subgroup analysis of included randomized controlled trials in the meta-analysis of ACV and FBS in T2DM patients.

Variables	Dose (ml/d)		Duration (weeks)		Design	
FBS (mg/dl)	≤ 15	< 8	< 8	≥8	B	NB
Number of comparisons	4	2	2	5	3	4
WMD (95% CI)	−15.66 (−24.12, −7.19)	−18.12 (−29.11, −7.12)	−18.12 (−29.11, −7.12)	−24.34 (−31.73, −16.96)	−16.42 (−25.29, −7.55)	−27.89 (−36.38, −19.41)
*p*-value	< 0.001	0.001	0.001	< 0.001	< 0.001	< 0.001
P between	0.02		0.36		0.07	
I^2^ (%)	0	0	0	37.38	0	17.1
P-heterogeneity	0.72	0.38	0.38	0.17	0.6	0.31

FBS, fasting blood sugar; B, blinded; NB, Not-blinded.

Various results achieved from this part could cause uncertainty regarding the association between dose of ACV and FBS changes and needs further evaluations.

#### 3.4.2 Meta-analysis on ACV and HbA1c

We included 4 studies and 319 participants in the HbA1c analysis. ACV significantly reduced HbA1c based on the results of a random-effect model (WMD: −1.53, 95% CI: −2.65, −0.41, *p* = 0.008), with significant heterogeneity (I^2^ = 83.31%, *p* < 0.001) (Figure 2B).

#### 3.4.3 Meta-analysis on ACV and HOMA-IR

The present study included 3 studies and 173 participants in the HOMA-IR analysis. ACV did not influence HOMA-IR significantly (WMD: 0.631, 95% CI: −0.99, 2.25, *p* = 0.446), and the studies’ heterogeneity was not significant (I^2^ = 56.2%, *p* = 0.102) (Figure 2C).

#### 3.4.4 Meta-analysis on ACV and insulin

We included 3 studies and 173 participants in the serum insulin analysis. ACV increased insulin levels based on the results of a random-effect model (WMD: 2.059 μu/ml, 95% CI: 0.26, 3.86, *p* = 0.025), with non-significant heterogeneity (I^2^ = 0%, *p* = 0.42) (Figure 2D).

### 3.5 Sensitivity analysis

As shown in the leave-one-out sensitivity analysis, the effect sizes of ACV on FBS and HbA1c were robust, indicating that the removal of every trial had no significant impact on the meta-analysis results (Figures S1A,B). In spite of this, the effect of ACV on HOMA-IR, and insulin was sensitive to one (25) and two (24, 27) studies, respectively (Figures S1C,D).

### 3.6 Publication bias

According to the “trim and fill” method, for FBS, HbA1c, HOMA-IR and insulin there were 1, 0, 1 and 2 studies that were missing, respectively (Figure S3A,D). We summarized the corrected effect sizes and the results of Begg’s rank correlation, Egger’s linear regression, and “fail-safe N” tests in Table S2.

## 4 Discussion

This systematic review and meta-analysis reviewed the available literature and CTs assessing the effects of ACV on glycemic factors and insulin sensitivity in T2DM. ACV could significantly reduce FBS and HbA1c levels. However, ACV increased insulin levels. Based on the dose–response analysis, the present study found linear and non-linear associations between ACV doses and FBS levels. The present study showed significant reductions in doses >10 mL/dL.

Hence, the current study showed decreasing trends in FBS that were in line with that of a meta-analysis reporting the reducing effects of ACV on fasting plasma glucose (FPG) in individuals with diabetes, overweight, or obesity (31). However, they did not show any relationship between the dose and duration of the supplementation of ACV with the changes in FPG (31) which was different from the result of the present study focusing on the effects of ACV dose on the FBS changes. The main reason for this difference could be possibly due to the differences in the included population and their baseline FPG. The present study only included patients with T2DM, while in that study (31), they included non-diabetic patients as well. They also emphasized the glucose-lowering effects of ACV in patients with diabetes rather than the no-diabetic ones in their sub-group analysis. They emphasized that higher baseline FPG could cause better results following ACV supplementation (49). This could almost justify better results in higher doses of ACV in patients with diabetes as in the present study. On the other hand, in accordance with the present finding, in another meta-analysis, vinegar consumption could decrease FBS as an important cardio-metabolic factor (49).

It is important to note that, there are several mechanisms for justifying the effects of ACV on glycemic control and improving FBS concentrations. ACV could cause a delay in gastric emptying and could improve the utilization of glucose. On the other hand, ACV could decrease liver glucose production and enhance the secretion of insulin (50, 51). Acetic acid content of ACV could inhibit disaccharidase (52) and *α*-amylase (21). This way, it can consequently decrease blood glucose. This mechanism can also explain the glucose-lowering effects of ACV. Further, increases in hepatic and muscle uptake of glucose could happen following ACV consumption and this could also explain hypoglycemic effects of ACV. On the other hand, ACV could increase the activity of glycogen synthase and decrease glycolysis. It was observed that acetic acid could increase glycogen repletion and this could also affect glucose uptake (53–55) and these could justify the possible role of ACV containing acetic acid in glycemic control. It was hypothesized that acetate metabolism through tricarboxylic acid cycle via acetyl-CoA (56) which can obtain acetate, could also affect glycogen synthase activation in the liver (57). All of these could help blood glucose control as well. However, these are mechanisms which have been explored, otherwise it remains a mere speculation. Moreover, one of the main polyphenols named chlorogenic acid present in ACV could cause glucose-6-phosphatase inhibition in rats. This can in turn decrease glucose release in the process of gluconeogenesis and glycogenolysis and decrease blood glucose consequently (58). It is clear that higher doses of ACV could exert more beneficial effects due to the higher active and effective components. All the aforementioned mechanisms could explain and justify the decreasing trend of FBS following ACV consumption. These effects can be more pronounced in the higher doses. However, further researches are warranted to better elucidate the exact dose of ACV with the maximum glucose-lowering effects.

In addition, as another finding, considering the results of ACV and HbA1C, we have seen a significant reduction in HbA1C following ACV consumption. However, the present study did not show any significant changes in the levels of insulin and HOMA-IR after ACV consumption. These results were in accordance with the results of the meta-analysis by Hadi et al. (31) that showed a decreasing trend in HbA1c after ACV consumption. However, they showed no changes in insulin or HOMA-IR. They also emphasized the beneficial effects of ACV on HbA1c in patients with diabetes with higher baseline FPG rather than non-diabetes ones (48). This was also seen in the current meta-analysis with the target population of patients with T2DM. This was also confirmed by two other meta-analyses which demonstrated that vinegar consumption could significantly decrease HbA1c (49, 59). HbA1c is considered a marker for glucose control in the past 2–3 months in patients with diabetes (52). Vinegar could ameliorate the insulin response to food glycemic index in patients with diabetes and could decrease HbA1c with this mechanism (60, 61). However, the current study showed increasing effects of ACV on insulin levels which seems unexpectable as is not in line with the results of other glycemic markers in the present meta-analysis. This finding was not in accordance with the finding of another meta-analysis by Shishehbor et al. (62) that mentioned the reducing trend in insulin levels following vinegar consumption. The main differences between that study and the present meta-analysis are related to the type of vinegar and the included studies containing insulin data (8 trials in their studies vs. three trials in the current study). On the other hand, in another meta-analysis by Hadi et al., no significant changes were seen in insulin concentration following ACV consumption in adults (31). Also, in a meta-analysis in 2022, vinegar consumption did not change serum insulin in healthy individuals and in those with cardio-metabolic diseases (49). Moreover, it is noteworthy to state that we included a small number of studies for assessing the effects of ACV on insulin and HOMA-IR (two for HOMA-IR and three for insulin). This cause uncertainty for drawing reliable conclusions in this regard. In addition, this issue was also mentioned in the study by Hadi et al. (31) as they included a few studies for these variables. Hence, the results considering the effects of ACV on insulin and HOMA-IR should be interpreted with caution. More investigations are warranted. However, a promising effect for HbA1c was observed.

Finally, regarding the sensitivity analysis, for all parameters, the removal of any study did not affect the results, except for HbA1c which was sensitive to two studies (28, 30) and insulin which was sensitive to one study that showed decreasing trends in insulin levels following ACV consumption in type 2 hyperlipidemic patients (25). Moreover, as we included only 3 studies for assessing the effects of ACV on insulin levels, the opposite effect of one study on the results could cause uncertainty regarding the increasing effects of ACV on insulin levels. On the other hand, the two studies affecting the results of HbA1c are those that showed promising effects of ACV on HbA1c. This could avoid reliable and definite conclusions to be drawn in this regard. Hence, the results of ACV on HbA1c and insulin should be interpreted with caution.

The present systematic review and meta-analysis pooled the available literature (CTs) assessing the effects of ACV on glycemic control and insulin sensitivity. This study had some limitations and strengths. As a limitation, we included the small number of studies for some variables such as insulin and HOMA-IR which could cause uncertainty regarding the final conclusions for those parameters. Also, we could not conduct sub-group analysis or meta-regression conduction for those variables. On the other hand, from seven included studies, five were conducted in Iran and totally 6 studies were in Asia (five in Iran and one in Pakistan) and this could affect the final interpretation of results and the results could not be possibly generalized to all populations in various geographical locations. Hence, interpretation of the final results should be done with caution. However, as a strength, meta-regression and sub-group analysis (based on dose, design, and duration of the studies) was done for FBS which could cause better definite results. Moreover, linear and non-linear dose–response relationships between the FBS parameter and ACV dosage were examined. Another strength is that we evaluated the ACV effects on a specific population (T2DM) in this meta-analysis. Also, as another strength, we observed non-significant heterogeneity among the included studies with most of the assessed parameters which could cause better uniformity among them. Hence, the final results could be possibly more reliable in this study from this point of view.

## 5 Conclusion

To sum up, the present systematic review and meta-analysis showed promising effects of ACV on FBS with a dose–response effect in patients with T2DM. The effects of ACV on decreasing HbA1c and increasing insulin were not definite due to the effects of omissions of two studies that could possibly change the results of HbA1c and one study that could affect insulin and the small number of studies included in insulin assessment. Moreover, we observed no changes in insulin resistance parameter including hemostatic model assessment for insulin resistance (HOMA-IR) following ACV consumption. This could be pertinent to the small number of studies that we included in this regard. Finally, it can be mentioned that further investigations are needed to better elucidate the exact effects of ACV on insulin, HOMA-IR, and HbA1c and to better definite the best effective dose of ACV with glucose-lowering effects, especially in various populations.

## Data Availability

The original contributions presented in the study are included in the article/Supplementary material, further inquiries can be directed to the corresponding author.
