# Supplementary material for: Effects of apple cider vinegar on glycemic control and insulin sensitivity in patients with type 2 diabetes: A GRADE-assessed systematic review and dose–response meta-analysis of controlled clinical trials
Source: Front Nutr. 2025 Jan 30;12:1528383. doi: 10.3389/fnut.2025.1528383 (PMC11821484; doi:10.3389/fnut.2025.1528383)
Supplement: Supplementary file 1 [file Data_Sheet_1.docx]

**Title page:**

**Effects of apple cider vinegar on glycemic control and insulin sensitivity in patients with type 2 diabetes: A GRADE-assessed systematic review and dose-response meta-analysis of controlled clinical trials**

Donya Arjmandfard ^1^ (MSc), Mehrdad Behzadi ^1^ (MSc), Zahra Sohrabi ^2, *^ (PhD), Mohsen Mohammadi Sartang ^2, *^ (PhD)

^1^ Student Research Committee, School of Nutrition and Food Sciences, Shiraz University of Medical Sciences, Shiraz, Iran

^2^ Nutrition Research Center, School of Nutrition and Food Sciences, Shiraz University of Medical Sciences, Shiraz, Iran

*** Corresponding authors:**

These authors were equally involved in the current study.

Zahra Sohrabi

Mohsen Mohammadi Sartang

Address: Department of Clinical Nutrition, School of Nutrition and Food Sciences, Shiraz University of Medical Sciences, Razi Blvd, Shiraz, Iran.

Fax Number: +98 37257288

Email: [Zahra_2043@yahoo.com](mailto:Zahra_2043@yahoo.com), [dr.mohamadi_nut@yahoo.com](mailto:dr.mohamadi_nut@yahoo.com)

**Data described in the manuscript will be made available upon request pending application and approval by contacting the Corresponding author.**

**(A)**

**(B)**

**(C)**

**(D)**

Figure s1. A-D. The results of sensitivity analysis for WMD analysis of FBS (A), HbA1c (B), HOMA-IR (C) and insulin (D).

**(A)**

**(B)**

Figure s2. A, B. Meta-regression between changes in FBS, and administered doses and intervention duration of ACV. A: FBS dose, B: FBS duration.

(A)

(B)

(C)

(D)

Figure s3. A, B. Assessment of publication bias in the impact of ACV on A: FBS, B: HbA1c, C: HOMA-IR, and D: insulin in T2DM patients.

**Table s1. Search strategy in databases**

|  | SEARCH STRATEGY | NUMBER |
| --- | --- | --- |
| PUBMED | ("malus"[MeSH Terms] OR "Malus domestica"[Title/Abstract] OR "Rosacea"[MeSH Terms] OR "trees apple"[Title/Abstract] OR "tree apple"[Title/Abstract] OR "apple crab"[Title/Abstract] OR "crab apple*"[Title/Abstract] OR "apple*"[Title/Abstract]) AND ("diabetes mellitus*"[MeSH Terms] OR "diabetes mellitus, type 2"[MeSH Terms] OR "type 2 diabetes mellitus"[Title/Abstract] OR "T2DM"[Title/Abstract] OR "diabetes mellitus type II"[Title/Abstract] OR "T2D"[Title/Abstract] OR "dexlipotam"[Supplementary Concept] OR "Patients with diabetes"[Title/Abstract] OR "diabetes type 2"[Title/Abstract] OR "type 2 diabetes"[Title/Abstract] OR "diabetes*"[Title/Abstract] OR "diabetes Complications"[MeSH Terms] OR "diabetes mellitus type 2"[Title/Abstract] OR "NIDDM"[Title/Abstract] OR "noninsulin dependent diabetes mellitus"[Title/Abstract] OR "Ketosis-Resistant Diabetes Mellitus"[Title/Abstract] OR "Stable Diabetes Mellitus"[Title/Abstract] OR "maturity onset diabetes mellitus"[Title/Abstract] OR "maturity onset diabetes mellitus"[Title/Abstract] OR "MODY"[Title/Abstract] OR "type 2 diabetes mellitus"[Title/Abstract] OR "maturity onset diabetes"[Title/Abstract] OR "diabetes maturity onset"[Title/Abstract] OR "maturity onset diabetes"[Title/Abstract] OR "type II diabetes"[Title/Abstract] OR "type 2 diabetes"[Title/Abstract] OR "diabetes mellitus type 2"[Title/Abstract] OR "diabetes mellitus type II"[Title/Abstract] OR "type II diabetes mellitus"[Title/Abstract] OR "DM2"[Title/Abstract] OR "diabetes mellitus adult onset"[Title/Abstract] OR "Adult-Onset Diabetes Mellitus"[Title/Abstract] OR "diabetes mellitus adult onset"[Title/Abstract] OR "lipoatrophic diabetes*"[Title/Abstract] OR "diabetes lipoatrophic"[Title/Abstract] OR "noninsulin dependent diabetes mellitus"[Title/Abstract]) AND ("randomized controlled trial"[Publication Type] OR "controlled clinical trial"[Publication Type] OR "random"[Title/Abstract] OR "randomly"[Title/Abstract] OR "clinical trial"[Publication Type] OR "controlled trial"[Title/Abstract] OR "Randomized"[Title/Abstract] OR "Randomised"[Title/Abstract] OR "clinical"[Title/Abstract] OR "placebo"[Title/Abstract] OR "trial"[Text Word] OR "clinical trial*"[Title/Abstract] OR "controlled trial*"[Title/Abstract] OR "intervention"[Title/Abstract] OR "Follow Up Studies"[MeSH Terms] OR "Comparative Study"[Publication Type] OR "cross-over trial"[Title/Abstract] OR "Cross-Over Studies"[Title/Abstract] OR "cross-over"[Text Word] OR "Random Allocation"[MeSH Terms] OR "Single-Blind Method"[MeSH Terms] OR "Double-Blind Method"[MeSH Terms] OR "Double-Blind"[Title/Abstract] OR "single-blind"[Title/Abstract] OR "assignment"[Text Word] OR "RCT"[Title/Abstract] OR "parallel"[Text Word]) | 142 |
| Scopus | ( TITLE-ABS-KEY ( "apple*" ) OR TITLE-ABS-KEY ( "crab apple*" ) OR TITLE-ABS-KEY ( "apple, crab" ) OR TITLE-ABS-KEY ( "apples, crab" ) OR TITLE-ABS-KEY ( "tree, apple" ) OR TITLE-ABS-KEY ( "trees, apple" ) OR TITLE-ABS-KEY ( "rosacea" ) OR TITLE-ABS-KEY ( "malus domestica" ) OR TITLE-ABS-KEY ( "malus*" ) ) AND ( TITLE-ABS-KEY ( "glycemic control" ) OR TITLE-ABS-KEY ( "glycemic index*" ) OR TITLE-ABS-KEY ( "glucose intolerance" ) OR TITLE-ABS-KEY ( "glucose tolerance tests" ) OR TITLE-ABS-KEY ( "oral glucose tolerance test" ) OR TITLE-ABS-KEY ( "ogtt" ) OR TITLE-ABS-KEY ( "glucose metabolism" ) OR TITLE-ABS-KEY ( "hyperglycemia" ) OR TITLE-ABS-KEY ( "glucose*" ) OR TITLE-ABS-KEY ( "glycemic*" ) OR TITLE-ABS-KEY ( "hba1c" ) OR TITLE-ABS-KEY ( "hemoglobin a1c" ) OR TITLE-ABS-KEY ( "glycated hemoglobin" ) OR TITLE-ABS-KEY ( "homa-ir" ) OR TITLE-ABS-KEY ( "homa ir" ) OR TITLE-ABS-KEY ( "quicki" ) OR TITLE-ABS-KEY ( "insulin" ) OR TITLE-ABS-KEY ( "insulin resistance" ) OR TITLE-ABS-KEY ( "insulin sensitivity" ) OR TITLE-ABS-KEY ( "glucose homeostasis" ) OR TITLE-ABS-KEY ( "insulin secretion" ) OR TITLE-ABS-KEY ( "glycemic indices" ) OR TITLE-ABS-KEY ( "blood glucose" ) OR TITLE-ABS-KEY ( "blood sugar" ) OR TITLE-ABS-KEY ( "fbs" ) OR TITLE-ABS-KEY ( "fpg" ) OR TITLE-ABS-KEY ( "fasting blood sugar" ) OR TITLE-ABS-KEY ( "fasting plasma glucose" ) OR TITLE-ABS-KEY ( "fasting glucose" ) OR TITLE-ABS-KEY ( "impaired fasting glucose" ) OR TITLE-ABS-KEY ( "homeostasis model assessment" ) OR TITLE-ABS-KEY ( "control, glycemic" ) OR TITLE-ABS-KEY ( "blood glucose control" ) OR TITLE-ABS-KEY ( "glycemic indices" ) OR TITLE-ABS-KEY ( "control, blood glucose" ) OR TITLE-ABS-KEY ( "Glucose Control, Blood" ) ) AND ( TITLE-ABS-KEY ( "t2dm" ) OR TITLE-ABS-KEY ( "diabetes mellitus*" ) OR TITLE-ABS-KEY ( "type 2 diabetes mellitus" ) OR TITLE-ABS-KEY ( "diabetes mellitus, type ii" ) OR TITLE-ABS-KEY ( "t2d" ) OR TITLE-ABS-KEY ( "patients with diabetes" ) OR TITLE-ABS-KEY ( "diabetes, type 2" ) OR TITLE-ABS-KEY ( "type 2 diabetes" ) OR TITLE-ABS-KEY ( "diabetes complications" ) OR TITLE-ABS-KEY ( "diabetes mellitus, type 2" ) OR TITLE-ABS-KEY ( "diabetic" ) OR TITLE-ABS-KEY ( "niddm" ) OR TITLE-ABS-KEY ( "noninsulin-dependent diabetes mellitus" ) OR TITLE-ABS-KEY ( "ketosis-resistant diabetes mellitus" ) OR TITLE-ABS-KEY ( "stable diabetes mellitus" ) OR TITLE-ABS-KEY ( "maturity-onset diabetes mellitus" ) OR TITLE-ABS-KEY ( "maturity onset diabetes mellitus" ) OR TITLE-ABS-KEY ( "mody" ) OR TITLE-ABS-KEY ( "slow-onset diabetes mellitus" ) OR TITLE-ABS-KEY ( "type 2 diabetes mellitus" ) OR TITLE-ABS-KEY ( "maturity-onset diabetes" ) OR TITLE-ABS-KEY ( "diabetes, maturity-onset" ) OR TITLE-ABS-KEY ( "maturity onset diabetes" ) OR TITLE-ABS-KEY ( "type ii diabetes" ) OR TITLE-ABS-KEY ( "type 2 diabetes" ) OR TITLE-ABS-KEY ( "diabetes*" ) OR TITLE-ABS-KEY ( "diabetes mellitus type 2" ) OR TITLE-ABS-KEY ( "diabetes mellitus type ii" ) OR TITLE-ABS-KEY ( "type ii diabetes mellitus" ) OR TITLE-ABS-KEY ( "dm2" ) OR TITLE-ABS-KEY ( "Diabetes Mellitus, Adult-Onset" ) OR TITLE-ABS-KEY ( "Adult-Onset Diabetes Mellitus" ) OR TITLE-ABS-KEY ( "Diabetes Mellitus, Adult Onset" ) OR TITLE-ABS-KEY ( "Lipoatrophic Diabetes*" ) OR TITLE-ABS-KEY ( "Diabetes, Lipoatrophic" ) OR TITLE-ABS-KEY ( "Noninsulin Dependent Diabetes Mellitus" ) ) AND ( TITLE-ABS-KEY ( "randomized controlled trial" ) OR TITLE-ABS-KEY ( "controlled clinical trial" ) OR TITLE-ABS-KEY ( "random" ) OR TITLE-ABS-KEY ( "randomly" ) OR TITLE-ABS-KEY ( "clinical trial" ) OR TITLE-ABS-KEY ( "controlled trial" ) OR TITLE-ABS-KEY ( "randomized" ) OR TITLE-ABS-KEY ( "clinical" ) OR TITLE-ABS-KEY ( "placebo" ) OR TITLE-ABS-KEY ( "trial" ) OR TITLE-ABS-KEY ( "clinical trial*" ) OR TITLE-ABS-KEY ( "controlled trial*" ) OR TITLE-ABS-KEY ( "intervention" ) OR TITLE-ABS-KEY ( "follow- up studies" ) OR TITLE-ABS-KEY ( "comparative study" ) OR TITLE-ABS-KEY ( "cross-over trial" ) OR TITLE-ABS-KEY ( "cross-over studies" ) OR TITLE-ABS-KEY ( "clinical trial as topic" ) OR TITLE-ABS-KEY ( "random allocation" ) OR TITLE-ABS-KEY ( "single-blind method" ) OR TITLE-ABS-KEY ( "double-blind method" ) OR TITLE-ABS-KEY ( "double-blind" ) OR TITLE-ABS-KEY ( "single-blind" ) OR TITLE-ABS-KEY ( "assignment" ) OR TITLE-ABS-KEY ( "rct" ) OR TITLE-ABS-KEY ( "parallel" ) OR TITLE-ABS-KEY ( "cross-over" ) OR TITLE-ABS-KEY ( "randomised" ) ) | 294 |
| ISI web of sciences | 1- “Apple*” (Topic) or “Crab Apple*” (Topic) or “Apple, Crab” (Topic) or “Apples, Crab” (Topic) or “Tree, Apple” (Topic) or “Trees, Apple” (Topic) or “Rosacea” (Topic) or “Malus domestica” (Topic) or “Malus*” (Topic)  2- “glycemic control” (Topic) or “Glycemic Index*” (Topic) or “glucose intolerance” (Topic) or “glucose tolerance tests” (Topic) or “oral glucose tolerance test” (Topic) or “OGTT” (Topic) or “glucose metabolism” (Topic) or “hyperglycemia” (Topic) or “glucose*” (Topic) or “GLYCEMIC*” (Topic) or “HbA1c” (Topic) or “hemoglobin A1C” (Topic) or “glycated hemoglobin” (Topic) or “HOMA-IR” (Topic) or “HOMA IR” (Topic) or “QUICKI” (Topic) or “insulin” (Topic) or “insulin resistance” (Topic) or “insulin sensitivity” (Topic) or “glucose homeostasis” (Topic) or “insulin secretion” (Topic) or “Glycemic Indices” (Topic) or “blood glucose” (Topic) or “blood sugar” (Topic) or “FBS” (Topic) or “FPG” (Topic) or “fasting blood sugar” (Topic) or “fasting plasma glucose” (Topic) or “fasting glucose” (Topic) or “impaired fasting glucose” (Topic) or “homeostasis model assessment” (Topic) or “Control, Glycemic” (Topic) or “Blood Glucose control” (Topic) or “Control, Blood Glucose” (Topic) or “Glucose Control, Blood” (Topic) or “Glycemic Indices” (Topic)  3- “T2DM” (Topic) or “diabetes mellitus*” (Topic) or “type 2 diabetes Mellitus” (Topic) or “diabetes mellitus, type II” (Topic) or “T2D” (Topic) or “Patients with diabetes” (Topic) or “Diabetes, Type 2” (Topic) or “type 2 diabetes” (Topic) or “diabetes*” (Topic) or “diabetes Complications” (Topic) or “diabetes mellitus, type 2” (Topic) or “diabetic” (Topic) or “NIDDM” (Topic) or “noninsulin-dependent diabetes mellitus” (Topic) or “Ketosis-Resistant Diabetes Mellitus” (Topic) or “Stable Diabetes Mellitus” (Topic) or “Maturity-Onset Diabetes Mellitus” (Topic) or “Maturity Onset Diabetes Mellitus” (Topic) or “MODY” (Topic) or “Slow-Onset Diabetes Mellitus” (Topic) or “Type 2 Diabetes Mellitus” (Topic) or “Maturity-Onset Diabetes” (Topic) or “Diabetes, Maturity-Onset” (Topic) or “Maturity Onset Diabetes” (Topic) or “type II diabetes” (Topic) or “type 2 diabetes” (Topic) or “diabetes mellitus type 2” (Topic) or “diabetes mellitus type II” (Topic) or “type II diabetes mellitus” (Topic) or “Adult-Onset Diabetes Mellitus” (Topic) or “DM2” (Topic) or “Diabetes Mellitus, Adult-Onset” (Topic) or “Diabetes Mellitus, Adult Onset” (Topic) or “Lipoatrophic Diabetes*” (Topic) or “Diabete, Lipoatrophic” (Topic) or “Diabetes, Lipoatrophic” (Topic) or “Lipoatrophic Diabete" (Topic) or "Noninsulin Dependent Diabetes Mellitus" (Topic)  4- “randomized controlled trial” (Topic) or “controlled clinical trial” (Topic) or “random” (Topic) or “randomly” (Topic) or “clinical trial” (Topic) or “controlled trial” (Topic) or “Randomized” (Topic) or “Randomised” (Topic) or “clinical” (Topic) or “placebo” (Topic) or “trial” (Topic) or “clinical trial*” (Topic) or “controlled trial*” (Topic) or “intervention” (Topic) or “Follow- UP Studies” (Topic) or “Comparative Study” (Topic) or “cross-over trial” (Topic) or “Cross-Over Studies” (Topic) or “Clinical Trial As Topic” (Topic) or “Random Allocation” (Topic) or “Single-Blind Method” (Topic) or “Double-Blind Method” (Topic) or “Double-Blind” (Topic) or “single-blind” (Topic) or “assignment” (Topic) or “RCT” (Topic) or “parallel” (Topic) or “cross-over” (Topic)  5- #4 AND #3 AND #2 AND #1 | 81 |

**Table s2. Assessment of publication bias in the impact of ACV on glycemic control.**

|  | Corrected effect size | | Begg's rank correlation test | | | Egger's linear regression test | | | | | Fail-safe N test |
| --- | --- | --- | --- | --- | --- | --- | --- | --- | --- | --- | --- |
|  | WMD | 95% CI | Kendall's Tau | z-value | p-value | Intercept | 95% CI | t | df | p-value | N |
| FBS | -23.09 | -17.1, -22.81 | 0.286 | 0.90 | 0.368 | 1.99 | -2.33,6.32 | 1.19 | 5 | 0.23 | 73 |
| HbA1c | - | - | 0 | 0 | 1 | 1.37 | -12.86,15.59 | 0.41 | 1 | 0.72 | 53 |
| HOMA-IR | 0.40 | -1.21, 2.15 | 0 | 0 | 1 | 1.02 | -30.75,32.8 | 0.41 | 1 | 0.75 | 0 |
| Insulin | 2.78 | 0.86, 4.7 | 0 | 0 | 1 | -0.85 | -17.43,15.74 | 0.65 | 1 | 0.63 | 0 |

Abbreviations: FBS: Fasting Blood Sugar, glycated hemoglobin (HbA1c), HOMA-IR: homeostasis model assessment for insulin resistance.
